# Rapid-onset type 1 diabetes mellitus and diabetic ketoacidosis: A case series of programmed cell death 1 inhibitor-associated endocrine toxicity in China

**DOI:** 10.1097/MD.0000000000051420

**Published:** 2026-10-30

**Authors:** Huihui Mao, Xuehui Jiang, Xiaowei Huang, Jingwen Fang, Yanling Mao, Lei Hong

**Affiliations:** a Department of Pharmacy, Quanzhou Traditional Chinese Medicine Hospital, Quanzhou, China; b Department of Pharmacy, Quanzhou First Hospital Affiliated to Fujian Medical University, Quanzhou, China; c Department of Endocrinology, Quanzhou First Hospital Affiliated to Fujian Medical University, Quanzhou, China.

**Keywords:** diabetic ketoacidosis, fulminant type 1 diabetes, immune checkpoint inhibitors, programmed death-1 inhibitor, type 1 diabetes mellitus

## Abstract

**Rationale:**

Immune checkpoint inhibitors (ICIs), including those targeting the programmed cell death 1 (PD-1) receptor, its ligand (PD-L1), and cytotoxic T lymphocyte antigen 4 (CTLA-4), are widely used in treating various cancers. Although generally well-tolerated, ICIs can cause severe immune-related adverse events (irAEs), including endocrine toxicities like hypophysitis, thyroid dysfunction, and adrenal insufficiency. Among these, ICI-induced type 1 diabetes mellitus (T1DM) is a rare but life-threatening irAE.

**Patient concerns:**

In order to provide a more comprehensive understanding of this condition, we describe 4 cases of PD-1 inhibitor-induced T1DM in Chinese patients.

**Diagnosis:**

All 4 patients developed diabetic ketoacidosis (DKA) within 1 week of hyperglycemic symptom onset, and 3 were diagnosed with fulminant T1DM. All patients were male, with a mean age of 63 years. The median time from PD-1 inhibitor initiation (sintilimab or camrelizumab) to T1DM diagnosis was 112 days. At onset, the mean blood glucose level was 684 mg/dL, mean glycated hemoglobin (HbA1c) was 8.22%, and mean serum C-peptide level was 0.13 ng/ml.

**Intervention:**

Intravenous insulin was transitioned to subcutaneous insulin in all patients, in addition to receiving fluid resuscitation and corrective electrolyte therapy.

**Outcomes:**

All patients eventually discontinued PD-1 inhibitor therapy. Through insulin treatment, their hyperglycemia was effectively controlled. Additionally, all our patients achieved stable disease or partial response in tumor treatment evaluation at T1DM onset.

**Lessons:**

These cases highlight that anti-PD-1 therapy can trigger a rapid and severe form of T1DM. All cancer patients receiving PD-1 inhibitors should undergo close monitoring of blood glucose levels and receive patient education.

## 1. Introduction

Immune checkpoint inhibitors (ICIs), including those targeting the programmed cell death 1 (PD-1) receptor, its ligand (PD-L1), and cytotoxic T lymphocyte antigen 4 (CTLA-4), are widely used in treating various cancers, such as metastatic melanoma, non-small cell lung cancer, and renal cell carcinoma. Although generally well-tolerated, ICIs can cause severe immune-related adverse events (irAEs), including endocrine toxicities like hypophysitis, thyroid dysfunction, and adrenal insufficiency.^[1]^ Among these, ICI-induced type 1 diabetes mellitus (T1DM) is a rare but serious irAE with an incidence of 0.2% to 1.4%,^[2,3]^ which often presents with life-threatening diabetic ketoacidosis (DKA). This adverse event occurs more frequently with PD-1/PD-L1 inhibitors than with CTLA-4 inhibitors.^[4-7]^ This difference may be attributed to the PD-1/PD-L1 pathway playing a more critical role in maintaining self-tolerance in pancreatic beta cells, coupled with the broader clinical use of PD-1/PD-L1 inhibitors. To further characterize this condition, we studied 4 Chinese patients who developed DKA following PD-1 inhibitor treatment in real-world practice, aiming to explore potential factors that might predispose patients to PD-1 inhibitor-related T1DM.

## 2. Patient case reports

### 2.1. Case 1

A 57-year-old Chinese man with a 6-month history of metastatic esophageal squamous cell carcinoma and synchronous sigmoid adenocarcinoma was initiated on a 21-day cycle of albumin-bound paclitaxel (300 mg), carboplatin (450 mg), and sintilimab (200 mg). His pretreatment glycemic parameters were within normal limits (HbA1c 5.3%; fasting blood glucose 83.34 mg/dL). After 9 cycles (approximately 180 days), he presented to the emergency department with a 4-day history of nausea, vomiting, dry mouth, and fatigue. Laboratory findings were consistent with diabetic ketoacidosis (DKA): blood glucose 433 mg/dL, ketones 6.5 mmol/L, pH 7.157, bicarbonate 9.0 mmol/L, and an elevated anion gap of 25.2. The diagnosis of DKA was established, and the patient was transferred to endocrinology for management with intravenous insulin, fluid resuscitation, and electrolyte correction. Further testing revealed an HbA1c of 8.3% and a low fasting C-peptide level of 0.16 ng/mL, with negative glutamic acid decarboxylase antibodies (GADA), insulin antibodies (IAA), and islet cell antibodies (ICA) results. His condition stabilized after transitioning to a subcutaneous basal-bolus insulin regimen, and he was subsequently discharged.

### 2.2. Case 2

A 61-year-old Chinese man with a 3-month history of poorly differentiated adenocarcinoma of the abdominal cavity began treatment. His pretreatment glycemic parameters were generally within normal limits (HbA1c not measured; fasting blood glucose 68.40 mg/dL). He received 1 cycle of sintilimab, oxaliplatin, and capecitabine, followed by 4 cycles of oxaliplatin, raltitrexed, and intraperitoneal fluorouracil. His blood glucose remained normal during this period. However, 72 days after the initiation of sintilimab, he presented to the emergency department with a 3-day history of dry mouth, fatigue, and poor appetite. Laboratory investigations revealed severe hyperglycemia (random blood glucose 786 mg/dL) with ketosis (blood ketones 6.4 mmol/L) and metabolic acidosis (pH 7.237, bicarbonate 12.4 mmol/L), leading to a diagnosis of diabetic ketoacidosis (DKA). Concurrently, the presence of a hyperosmolar state (serum osmolality 325 mOsm/L) supported the diagnosis of a combined hyperosmolar hyperglycemic state (HHS). The patient was transferred to the endocrinology department for intensive management. Further evaluation showed an HbA1c of 7.5%, a low fasting C-peptide level of 0.23 ng/mL, and negative β-cell autoantibodies (GADA, IAA, and ICA). After 12 days of hospitalization, during which his glycemic control was stabilized after transitioning from intravenous to a multiple daily subcutaneous insulin regimen, he was discharged.

### 2.3. Case 3

A 59-year-old Chinese man with a recent diagnosis of esophageal squamous carcinoma began a 21-day treatment cycle with camrelizumab plus platinum-paclitaxel chemotherapy. His baseline glycemic parameters were within normal limits (HbA1c 6.01%; fasting blood glucose 85.00 mg/dL). After 2 cycles (72 days), he presented to the emergency department with a 3-day history of dry mouth, polydipsia, vomiting, and fatigue. Laboratory evaluation confirmed diabetic ketoacidosis (DKA), evidenced by a blood glucose of 678 mg/dL, ketones of 7.0 mmol/L, pH 7.148, and bicarbonate of 10.8 mmol/L. Further workup revealed an HbA1c of 7.4%, a markedly low fasting C-peptide of 0.04 ng/mL, and a positive ICA result, whereas GADA and IAA were negative. Concurrently, the patient was diagnosed with clinical thyrotoxicosis (thyroid-stimulating hormone 0.006 μIU/mL, elevated fT3 and fT4) in the absence of thyroid peroxidase or thyroglobulin antibodies. Against medical advice, the patient was discharged prematurely prior to achieving glycemic control. Camrelizumab was resumed 1 week later, leading to a rapid recurrence of DKA with undetectable C-peptide (<0.02 ng/mL). The immunotherapy was subsequently discontinued at the patient’s request. Follow-up 2 months later confirmed the evolution of his thyroiditis from thyrotoxicosis to overt hypothyroidism (thyroid-stimulating hormone 26.01 μIU/mL, fT4 0.14 ng/dL), completing the classic clinical course of PD-1 inhibitor-induced thyroiditis.

### 2.4. Case 4

A 75-year-old Chinese man with metastatic esophageal cancer initiated treatment with camrelizumab. His baseline glycemic parameters were generally within normal limits (HbA1c not measured; fasting blood glucose 85.86 mg/dL). Five months (and 5 cycles, 146 days) later, he presented to the emergency department with a 4-day history of altered mental status. This occurred in the context of a prior, asymptomatic hyperglycemic episode (394 mg/dL) that had not required intervention. Laboratory findings on admission were critical, demonstrating a random blood glucose of 840 mg/dL, blood ketones of 7.4 mmol/L, metabolic acidosis (pH 7.083, bicarbonate 7.9 mmol/L), and elevated serum osmolality (325 mOsm/L), leading to a diagnosis of concurrent diabetic ketoacidosis (DKA) and hyperosmolar hyperglycemic state (HHS). Further evaluation revealed an HbA1c of 9.7%, a low fasting C-peptide of 0.1 ng/mL, and an elevated serum amylase (> 3 × upper limit) in the absence of radiographic evidence of pancreatitis. All tested pancreatic autoantibodies (GADA, IAA, ICA) were negative. Following intensive management with insulin and fluids, his hyperglycemia, ketosis, and hyperosmolar state resolved.

## 3. Ethic statement

The requirements for Institutional Review Board approval at Quanzhou First Hospital Affiliated to Fujian Medical University were waived due to the retrospective nature of this study. Written informed consent was obtained from all patients for publication of this case series.

## 4. Discussion

Type 1 diabetes mellitus induced by PD-1 inhibitors, though rare, can present as DKA and become life-threatening if not promptly treated. None of the 4 patients in this study had a history of diabetes prior to PD-1 inhibitor therapy. All were diagnosed with PD-1 inhibitor–induced T1DM based on random blood glucose > 200 mg/dL, ketosis with anion gap metabolic acidosis, and fasting C-peptide < 0.3 ng/mL. Potential alternative etiologies, including chemotherapy-related metabolic disturbances, occult type 2 diabetes mellitus, pancreatitis, pancreatic metastases, severe infection, corticosteroid exposure, and stress hyperglycemia secondary to advanced malignancy, were carefully considered. None of the patients received corticosteroids around the onset of hyperglycemia. There was no evidence of recent chemotherapy-related metabolic derangement or severe infection, and no clinical or radiographic evidence suggested pancreatitis or pancreatic metastases at diabetes onset. Stress-related hyperglycemia was considered unlikely given the markedly low fasting C-peptide levels (<0.3 ng/mL), consistent with severe insulin deficiency rather than transient insulin resistance. No prior clinical or laboratory evidence suggested occult type 2 diabetes or underlying pancreatic injury before PD-1 inhibitor initiation. Taken together, these clinical-laboratory findings supported the diagnosis of PD-1 inhibitor-induced T1DM.

Multiple case reports have described new-onset fulminant type 1 diabetes mellitus (FT1DM) following PD-1 inhibitor treatment,^[8-12]^ characterized by rapid hyperglycemia, ketoacidosis, absolute insulin deficiency, and near-normal HbA1c at presentation. Kyriacou et al reported that 43.1% of ICI-related T1DM patients met the modified Japanese criteria for FT1DM.^[13]^ A national survey in Japan found that FT1DM accounts for 50% of PD-1 inhibitor-related T1DM cases.^[14]^ In our series, cases 1 to 3 showed the characteristic FT1DM phenotype, with rapid development of diabetic ketoacidosis, marked hyperglycemia, HbA1c < 8.7%, and fasting C-peptide < 0.3 ng/mL. In contrast, case 4 had an HbA1c of 9.7% at presentation and documented hyperglycemia before the DKA episode and therefore did not strictly fulfill the classical FT1DM criteria. We therefore regard case 4 more conservatively as ICI-associated T1DM presenting with concurrent DKA and HHS rather than as definite FT1DM.^[15]^

Laboratory data and clinical characteristics of patients are summarized in Table 1. The clinical characteristics of our patients share similarities with previous reports,^[5,14,16]^ including a mean age of 63 years, a mean of 4.3 PD-1 inhibitor doses, and a median time to T1DM onset of 112 days. Notably, all patients in our cohort were male; however, given the small sample size (n = 4), this observation should be interpreted with caution and is hypothesis-generating rather than conclusive evidence of a sex predisposition, which awaits validation in larger cohorts. At presentation, the mean blood glucose was 684 mg/dL (range: 433–840 mg/dL), mean HbA1c was 8.22% (range: 7.4–9.7%), and mean fasting C-peptide was 0.13 ng/mL (range: 0.04–0.23 ng/mL). Serum C-peptide levels, reflecting β-cell function, were markedly low in all cases. All 4 patients developed DKA within 1 week, and 2 were concurrently diagnosed with HHS. Previous studies suggest that patients with combined DKA-HHS exhibit more severe hyperglycemia than those with DKA alone.^[17]^ In our cohort, the mean glucose level in the 2 patients with DKA-HHS was 813 mg/dL, whereas that in the 2 patients with DKA alone was 555 mg/dL. However, given that each subgroup contained only 2 cases, this numerical difference should not be interpreted as confirming the previously reported pattern, and any statistical or clinical comparison is inherently unreliable. These data await validation in larger series. Additionally, 2 patients with DKA-HHS developed acute kidney injury, consistent with reports of increased renal susceptibility in such cases.^[17]^ IrAEs may arise at any time during ICI therapy, including after treatment cessation.^[18]^ This is illustrated by one of our patients who developed T1DM 72 days after discontinuing PD-1 inhibitor therapy, underscoring that irAEs are not temporally restricted to the treatment phase. Furthermore, 1 patient developed thyroid-related irAEs while on camrelizumab, highlighting the potential for multi-organ immune toxicity.

**Table 1:** Laboratory data and characteristics of patients developing T1DM related to PD-1 inhibitors.

Case no.	1	2	3	4
Age (yr)	57	61	59	75
Sex	Male	Male	Male	Male
Weight (Kg)	69	65	53	53
Cancer subtype	Esophagus cancer, Colon cancer	Adenocarcinoma of the abdominal cavity	Esophagus cancer	Esophagus cancer
PD-1 inhibitor used	Sintilimab	Sintilimab	Camrelizumab	Camrelizumab
Associated chemotherapy	Carboplatin, albumin-bound paclitaxel	Oxaliplatin, raltitrexide, fluorouracil	Cisplatin, paclitaxel	None
Previous history of diabetes	No	No	No	No
Glucose (mg/dL)	433	786	678	840
Blood ketone	6.5	6.4	7.0	7.4
HbA1c (4.0–6.0%)	8.3	7.5	7.4	9.7
C-peptide (1.1–4.4 ng/mL)	0.16	0.23	0.04	0.1
2-hour postprandial C-peptide (ng/mL)	0.42	0.13	0.04	–
Serum pH (7.35–7.45)	7.157	7.237	7.148	7.083
Serum osmolarity (280–320 mOsm/L)	292	325	306	325
Serum bicarbonate (22–26 mmol/L)	9.0	12.4	10.8	7.9
Time from PD-1 inhibitor initiation to diagnosis				
Number of doses	9	1	2	5
Onset in days	180	72	45	150
Islet autoantibodies				
ICA	Negative	Negative	Positive	Negative
IAA	Negative	Negative	Negative	Negative
GADA	Negative	Negative	Negative	Negative
Other endocrinopathies				
Thyroid dysfunction	No	No	Yes	No
Adrenal insufficiency	No	No	No	No
Tumor response	Partial response	Stable disease	Stable disease	Stable disease
PD-1 inhibitor therapy after DKA	Stop	Stop	Stop	Stop
Amylase (35–135 U/L)	33	55	–	425
Lipase (≤67 U/L)	12	25	–	–
Average insulin dosage (U/Kg)	0.536	0.615	0.490	0.509

DKA = diabetic ketoacidosis, GADA = glutamic acid decarboxylase antibodies, IAA = insulin antibodies, ICA = islet cell antibodies, PD-1 = programmed death-1.

Most prior reports of PD-1 inhibitor-induced T1DM involve FDA-approved agents such as nivolumab and pembrolizumab.^[19-21]^ Combination therapy with anti-CTLA-4 and anti-PD-1/PD-L1 agents is known to increase the risk of ICI-induced T1DM.^[22-24]^ In our study, all patients received either sintilimab or camrelizumab. Among the tumor types represented, 3 had esophageal cancer and 1 had poorly differentiated abdominal adenocarcinoma. Whether certain cancer types predispose to T1DM as an irAE remains unclear due to the limited sample size.

Several islet autoantibodies have been implicated in ICI-related T1DM, including GADA, IAA, ICA, IA-2A, and ZnT8A. Clotman et al reported that 56% of PD-1-associated T1DM patients had positive islet autoantibodies, all of whom were GADA-positive, with 2 also testing positive for ICA.^[5]^ Another review of 42 cases found that 20 were GADA-positive, but none were ICA-positive.^[19]^ Conversely, Asian cohorts have reported substantially lower frequencies – for instance, Baden et al found only 4.8% GADA-positivity in a Japanese series^,[14]^ and Jin et al reported only 8.7% overall autoantibody positivity in a Chinese cohort – suggesting potential ethnic heterogeneity.^[25]^

Although our small sample precludes definitive conclusions, it is noteworthy that the sole autoantibody-positive patient in our cohort was ICA-positive, whereas GADA – the most prevalent antibody in Western series – was absent in all cases. We propose several explanations for this finding. First, ethnic differences in HLA backgrounds and autoantibody repertoires may influence the predominant humoral response; the lower GADA prevalence observed in Asian populations with ICI-T1DM aligns with this possibility. Second, the timing of antibody testing may influence antibody detectability, particularly in patients with rapidly evolving β-cell dysfunction. Third, inter-assay variability (e.g., ELISA, RIA, or electrochemiluminescence) and differing cutoff values across centers could affect reported positivity rates. Fourth, and most importantly, ICI-T1DM is primarily driven by T-cell-mediated immunity; autoreactive CD8^+^T cells – particularly IFN-γ-producing cytotoxic T cells – can directly destroy β-cells independently of detectable circulating autoantibodies,^[26]^ which may partly explain the high seronegativity rate reported in this condition, especially in fulminant presentations. Previous studies have suggested that autoantibody positivity may be associated with an earlier onset of ICI-related T1DM.^[7,27]^ However, given the very small sample size of our cohort, we did not attempt to draw conclusions regarding the relationship between autoantibody status and time to T1DM onset.

Certain human leukocyte antigen (HLA) genotypes, such as DR4 and DR9, are established risk factors for T1DM.^[28,29]^ Previous studies indicate that 67% of ICI-induced T1DM patients carry high-risk HLA genotypes,^[4]^ with HLA-DR4 present in up to 76% of cases.^[7]^ Specific haplotypes – including HLA-DRB1*04:05:01, DRB1*08:02:01, and DRB1*09:01-DQB1*03:03 – have been associated with increased ICI-T1DM risk,^[30]^ while others such as DRB1*15:02-DQB1*06:01 may confer protection.^[31]^ However, HLA testing does not capture all at-risk individuals and is often unavailable in routine practice, as was the case in our study. We acknowledge this as an important limitation. The absence of HLA genotyping substantially restricts our ability to assess whether the patients who developed ICI-T1DM in this cohort carried the known susceptibility alleles and therefore limits our ability to explore genetic predisposition in these cases. Given that HLA background is one of the strongest predictors of ICI-associated diabetes, this lack of data limits the generalizability of our findings and our ability to interpret the autoantibody and clinical findings within a genetic context.

The precise mechanism of PD-1 inhibitor-induced T1DM remains unclear, but it likely involves PD-1/PD-L1 pathway blockade leading to transcriptional immune alterations and enhanced T-cell infiltration into pancreatic islets.^[32]^ By inhibiting PD-1/PD-L1 binding, these agents reactivate antitumor T-cell activity.^[33]^ However, since PD-L1 is also expressed on islet cells, this may permit self-reactive T cells to attack β-cells, leading to profound insulin deficiency. Yet this simplified view does not fully explain the clinical observations. The fact that only a small minority of treated patients develop diabetes suggests that PD-1/PD-L1 blockade alone is insufficient; host susceptibility, including a preexisting pool of autoreactive T cells, the cytokine milieu, and interindividual differences in immune checkpoint expression or T-cell receptor repertoires, may influence disease development. The abrupt onset of β-cell destruction – often presenting as fulminant diabetes – may reflect rapid expansion of effector T cells and acute intra-islet cytotoxicity after checkpoint blockade rather than a slowly progressive autoimmune process; local inflammatory mediators and epitope spreading may further amplify this response.^[34]^ Genetic predisposition, particularly HLA risk alleles, may increase susceptibility, although cases also occur in patients without recognized high-risk genotypes, suggesting contributions from other genetic and environmental factors. Notably, a substantial proportion of patients are negative for conventional islet autoantibodies at diagnosis, which may reflect predominantly T-cell-mediated cytotoxicity, delayed antibody seroconversion, or limitations in antibody detection. Collectively, ICI-T1DM represents a distinct disease entity, in which checkpoint blockade interacts with individual genetic and immune susceptibility to influence both the likelihood and tempo of β-cell destruction.

The management of PD-1 inhibitor-induced T1DM with DKA follows conventional protocols, including intravenous fluids, continuous insulin infusion, and aggressive electrolyte repletion. These acute measures are consistent with recommendations from major oncology and endocrinology guidelines, such as those from ASCO, ESMO, and the ADA, which emphasize prompt recognition and standardized DKA care. However, guidelines also underscore the importance of preemptive screening: baseline fasting glucose and HbA1c should be obtained before initiating PD-1/PD-L1 inhibitors, particularly in patients with preexisting diabetes or risk factors. In cases 2 and 4, pretreatment fasting glucose was available, but baseline HbA1c had not been measured, indicating that baseline metabolic assessment was incomplete. During therapy, routine glucose monitoring is advised at each cycle or more frequently if symptoms arise, and ketone testing should be performed in patients with marked hyperglycemia or clinical suspicion of DKA.

Stamatouli et al reported a mean insulin requirement of 0.56 U/kg/d after diagnosis,^[7]^ which aligns closely with the average dosage in our cohort (0.54 U/kg/d). PD-1 inhibitor-associated T1DM is generally irreversible, with few reports of recovered islet function;^[35]^ none of our patients regained β‑cell function. Consequently, all affected patients face lifelong insulin dependence, necessitating structured diabetes education, frequent self-monitoring of blood glucose, and regular outpatient endocrinology follow-up. Although our short-term follow-up precluded detailed long-term glycemic assessment, it is critical to monitor post-discharge glycemic control (e.g., time-in-range, HbA1c) and to remain vigilant for recurrent DKA. In our series, one patient who resumed therapy despite suboptimal glycemic control developed recurrent DKA, highlighting that resolution of DKA and stable glycemic control should be achieved before rechallenge.^[36]^

Interestingly, some evidence suggests that immune-related adverse events (irAEs) may correlate with improved antitumor responses.^[37]^ All our patients achieved stable disease or partial response at T1DM onset, though the short follow-up limits assessment of this association. Long-term oncologic outcomes – such as progression-free and overall survival – remain to be evaluated in larger cohorts. PD-1 inhibitor-related T1DM is not an absolute contraindication to continuing immunotherapy; treatment may be resumed once glycemic control is achieved with insulin.^[38]^ In our series, all patients eventually discontinued PD-1 inhibitors, mostly due to patient preference, rather than the T1DM per se. Nonetheless, the decision to restart should be individualized, weighing anticancer benefit against the risk of metabolic decompensation.

Beyond acute management, long-term quality of life is a major concern. Patients require ongoing support from clinical pharmacists and diabetes educators to recognize hyperglycemic symptoms, manage insulin dosing, and prevent DKA. Multidisciplinary collaboration among oncologists, endocrinologists, and pharmacists is essential to optimize both metabolic and oncological outcomes. Thus, clinicians should maintain high awareness of irAEs, intensify glucose monitoring during PD-1 inhibitor therapy, and adopt a proactive, guideline-informed approach to screening, monitoring, and shared decision-making for rechallenge.

With immune checkpoint inhibitors playing a pivotal role in cancer treatment, it is crucial to enhance our comprehension of endocrine immune-related adverse events (irAEs), notably T1DM. Future mechanistic studies are needed to identify high-risk patients and prevent life-threatening complications such as DKA. There are several limitations in this retrospective case series. First, some islet autoantibodies such as IA-2 and ZnT8 antibodies were not detected owing to laboratory constraints, which may limit complete serological assessment. Second, HLA genotyping for susceptibility alleles was not performed. Further multi-center prospective studies in Chinese populations are required to better characterize autoantibody profiles and HLA-associated genetic susceptibility of ICI-associated type 1 diabetes mellitus.

## Author contributions

**Conceptualization:** Lei Hong.

**Data curation:** Huihui Mao, Xuehui Jiang, Xiaowei Huang, Jingwen Fang, Yanling Mao.

**Investigation:** Xuehui Jiang, Xiaowei Huang, Jingwen Fang, Yanling Mao.

**Writing – original draft:** Huihui Mao.

**Writing – review & editing:** Lei Hong.
